# Community knowledge and awareness of colorectal cancer and screening tools: Community-based survey of 1,912 residents of Riyadh

**DOI:** 10.1016/j.amsu.2021.103046

**Published:** 2021-11-14

**Authors:** Maram Abdullah Alaqel, Sulaiman Abdullah Alshammari, Shoag Mohammed Alahmari, Nawaf Khayal Alkhayal, Thamer Abdullah Bin Traiki, Noura Sufyan Alhassan, Omar Abdullah Al-Obeed, Ahmad Mohammed Zubaidi, Khayal Abdulmalik Alkhayal

**Affiliations:** a Colorectal Research Chair, Department of Surgery, College of Medicine, King Khalid University Hospital, King Saud University, Riyadh, Saudi Arabia; b College of Medicine, Alfaisal University, Riyadh, Saudi Arabia

**Keywords:** Cancer awareness, Cancer screening, Colonoscopy, Colorectal cancer, CT, Computerized tomography, CRC, colorectal cancer

## Abstract

**Background:**

Participation in Colorectal cancer (CRC) screening programs is low in Saudi Arabia. Public awareness of CRC and knowledge of available screening tools are crucial for improving screening uptake. This study aimed to examine the level of awareness and knowledge of CRC among the Saudi population.

**Materials and methods:**

A survey-based study was conducted on 1912 residents of Riyadh, Saudi Arabia. The survey comprised 20 questions; these concerned the definition of the colon and rectum; the function of the colon; the incidence, risk factors, symptoms, screening methods, prevention methods, and treatment methods for CRC; and the value of early detection of CRC.

**Results:**

Of the 1912 participants who completed the survey, only 51.7% knew that the colon was the large intestine, while 57% knew that the rectum was the end of the large intestine. Colonoscopy was the preferred screening tool (72.8%). Most respondents believed early detection of CRC through colonoscopy is associated with high survival rates. However, 65.7% of the participants reported that they would not like to undergo a CRC screening. Higher education level was also associated with knowledge that CRC can develop asymptomatically, with postgraduates most likely to know this (*P* = 0.032).

**Conclusions:**

There is a lack of knowledge regarding CRC among certain demographic groups in Saudi Arabia, and education and screening programs should target populations with the most limited knowledge.

## Highlights

• Colonoscopy was the preferred CRC-screening method among the sample.
• The biggest barrier to CRC screening was fear of diagnosis.
• Majority of respondents chose stress as stress factor for CRC.

## 1 Introduction

Colorectal cancer (CRC) is one of the most common malignancies worldwide, including in Saudi Arabia [1,2]. CRC is the second most common cancer among the Saudi population after breast cancer; the incidence of CRC among men and women with cancer is 14.2% and 9.3%, respectively [2]. It is the leading cancer among men in nine regions of Saudi Arabia, including Riyadh [2]. Patients older than 45 years are more likely to have CRC and usually present with advanced cancer stages [3]. Metastatic disease is the first presentation in 24% of Saudi patients, and the national death rate from CRC is approximately 8.3% [3,4]. The overall survival rate among patients in Saudi Arabia is 44.6%, which is lower than the approximately 60% reported in countries such as the US and Japan [5].

Globally, participation in CRC-screening programs is generally low [6]; in Saudi Arabia, such participation remains low despite high resource availability [7 8]. There is a direct relationship between screening uptake and CRC awareness [9]. Several Saudi-Arabia-based studies have reported low CRC awareness and screening uptake; most participants in these studies had no knowledge of colonoscopies, fecal occult blood tests, or computerized tomography (CT) scans [10,11]. Reasons for this low uptake of screening include a lack of physicians, distrust in Western medicine, and a belief that God determines an individual's destiny [12].

Public awareness of CRC and knowledge of available screening tools are crucial for improving screening uptake. This study aimed to examine the level of awareness and knowledge of CRC among the Saudi population. The data collected may contribute to the development of strategies for improving CRC-related knowledge among at-risk groups.

## 2 Methods

### 2.1 Sample and procedure

This survey-based study was conducted on 1912 residents of Riyadh, Saudi Arabia. The questionnaire, consent forms, and information leaflet were developed in Arabic, following a standard. The questionnaire was administered by volunteers from clinics in Riyadh, and the questions were phrased to maximize clarity. Subject recruitment was performed by approaching individuals in public shopping malls. The inclusion criterion was being 18 years of age or older; the exclusion criterion was a history of CRC or inflammatory bowel disease. All participants were supervised while they completed the survey, and questions were read aloud to those who were unable to read. The survey comprised 20 questions; these concerned the definition of the colon and rectum; the function of the colon; the incidence, risk factors, symptoms, screening methods, prevention methods, and treatment methods for CRC; and the value of early detection of CRC. All questions were in multiple-choice format, and multiple answers per question were permitted where appropriate. An information sheet provided with the questionnaire explained the study purpose and how to complete the survey. Anonymous personal data (age, gender, marital status, and education level) were also collected. For analyses, education level was grouped as follows: illiterate and primary school, intermediate school, secondary school, university, postgraduate, and involvement in medical school, respectively. Age was stratified by decade, as follows: 10–19, 20–29, 30–39, 40–49, 50–59, and 60+ years. Gender and marital status were also analyzed. The work has been reported in line with the STROCSS criteria [13]. The research registry UNI for this study is researchregistry7228 [14].

### 2.2 Ethical consideration

No personal identification information or other personal identifiers were recorded. The study protocol was approved by the ethical review board of [blinded for review].

### 2.3 Statistical analyses

SPSS version 26.0 (IBM Corp., Armonk, N·Y., USA) was used to perform univariate, bivariate, and stratified analyses of the data. Qualitative variables were analyzed by constructing contingency tables using Pearson's χ^2^ test, or Fisher's exact test when the conditions for the former were not met. *P* ≤ 0.05 was considered to indicate significance.

## 3 Results

The respondents’ demographic characteristics, including age, marital status, education level, medical background, and gender, are presented in Table 1. Overall, 1912 participants completed the survey. Most respondents were single (61.3%), female (73.1%), 20–29 years old (46.4%), with university education (72.2%), and no medical background (81%).

**Table 1:** Respondents’ demographic characteristics.

**Gender**	Male	514 (26.9)
	Female	1398 (73.1)
	Median (IQR)	25.0 (20.0–35.0)
**Age group**	<20	360 (18.80)
	20–29	888 (46.4)
	30–39	286 (15)
	40–49	207 (10.8)
	50–59	116 (6.1)
	≥60y	55 (2.9)
**Marital status**	Single	1172 (61.3)
	Married	672 (35.1)
	Divorced	49 (2.6)
	Widowed	19 (1)
**Educational level**	Illiterate	8 (0.40)
	Primary-secondary	350 (18.3)
	University	1380 (72.2)
	Post graduate	174 (9.1)
**Medical background**	Yes	363 (19)
	No	1549 (81)

IQR: Interquartile range.

Respondents answered the items shown in Table 2. The most common answer to the item ‘which type of cancer is associated with the most fatalities in Saudi Arabia?’ was colon cancer (56%). Only 51.7% knew that the colon was the large intestine, while 57% knew that the rectum was the end of the large intestine. Most respondents (61.9%) believed that stress is a risk factor for CRC. Regarding CRC symptoms, 44.6% knew that CRC could develop asymptomatically. Regarding the most common CRC-related symptoms, approximately half of the participants chose the presence of blood in stool, abdominal pain, and bloating, respectively (51.9%, 51.5%, and 51.2%, respectively).

**Table 2:** Data for correct and common responses to the questionnaire items.

Question	Correct response/Common responses	Number of respondents (n = 1912)	Percentage, %
**Which type of cancer is associated with the most fatalities in Saudi Arabia?**	Colon cancer	1070	56.0
**What is the colon?**	The large intestine	989	51.7
**What is the rectum?**	Last part of the large intestine	1089	57.0
**What is the function of the colon?**	Water reabsorption	407	21.3
**What are risk factors for CRC?**	Stress	1183	61.9
	Family history	962	50.3
	Smoking	773	40.4
**Can CRC develop asymptomatically?**	Yes	853	44.6
**What symptoms are associated with CRC?**	Presence of blood in stool	993	51.9
	Abdominal pain	984	51.5
	Bloating	979	51.2
	Change in bowel habits	873	45.7
	Nausea and vomiting	468	24.5
**Can CRC be prevented?**	Yes	1756	91.8
**Can CRC be cured if detected early?**	Yes	1862	97.4
**Would you undergo a colonoscopy if recommended to do so by a physician?**	Pay 2000 SR to undergo a colonoscopy in the near future	1117	58.4
	Wait for six months to receive a colonoscopy for free	687	35.9
	Would not undergo a colonoscopy	108	5.6
**Are you willing to undergo screening for CRC even without experiencing any symptoms?**	Yes	1327	69.4
**Preferred screening method**	Colonoscopy, detects 95% of cases	967	72.8
**Is early detection of CRC through a colonoscopy associated with high survival rates?**	Yes	1766	92.4
**Would you like to undergo CRC screening?**	Yes	1766	92.4
**Barriers to undergoing CRC screening**	Fear of diagnosis/denial	578	30.2
	Delays due to administerial reasons	511	26.7

CRC: Colorectal cancer.

Most respondents (91.8%) believed that CRC is preventable, and 97.4% thought that CRC could be cured if detected early. Most respondents believed that colonoscopies are important. In a hypothetical situation in which a physician recommended that the participant undergo a colonoscopy, and in which there was a choice between undergoing a colonoscopy immediately at a cost of 2000 SR or receiving a colonoscopy free of charge in six months, 58.4% chose the former while 35.9% chose the latter. Colonoscopy was the preferred CRC-screening method among the sample (72.8%).

Most respondents (92.4%) believed early detection of CRC through colonoscopy is associated with high survival rates. However, 65.7% of the participants reported that they would not like to undergo a CRC screening. The biggest barrier to CRC screening was fear of diagnosis (30.2%), followed by the possibility of delays due to administerial reasons (26.7%).

Differences in responses in terms of demographic groups (gender, age, education level, marital status, and medical background) are shown in Table 3.

**Table 3:** Differences in colorectal cancer knowledge among the general Saudi population in terms of gender, age, marital status, and education level.

Question		Gender	Age	Marital status	Education level
**Cancer type associated with most fatalities**		0.02*	0.759	0.475	0.124
**What is the colon?**		<0.001**	<0.001**	0.033*	0.005**
**What is the rectum?**		<0.001**	<0.001**	<0.001**	<0.001**
**Colon function**		<0.001**	<0.001**	<0.001**	0.002**
**Risk factors**	Age	0.004**	<0.001**	0.132	0.668
	Family history	<0.001**	<0.001**	0.051	0.068
	Smoking	0.239	0.031*	0.159	0.513
	Stress	0.287	<0.001**	<0.001**	0.515
**CRC symptoms**	Abdominal pain	0.382	<0.001**	<0.001**	0.138
	Change in bowel habits	<0.001**	0.002**	0.013*	0.001**
	Nausea and vomiting	<0.001**	0.022*	0.056	0.155
	Bloating	0.009**	<0.001**	<0.001**	0.263
	Presence of blood in stool	<0.001**	<0.001**	0.001**	<0.001**
	Don't know	0.001**	0.39	0.265	0.342
**CRC prevention**		0.061	0.586	0.635	0.905
**CRC cure**		0.25	0.496	0.181	0.256
**Colonoscopy**	Undergo now for 2000 SR or wait for six months	<0.001**	0.001**	0.231	0.328
**Screening test**		<0.001**	0.006**	0.637	0.057
**Preferred screening method**		<0.001**	<0.001**	<0.001**	0.037*
**Early detection and survival**		0.409	0.105	0.109	0.754
**Prefer CRC screening**		0.294	<0.001**	<0.001**	0.017*
**Barriers to CRC screening**		<0.001**	<0.001**	0.076	0.13

*P < 0.05.

Numbers are Pearson chi-square values.

CRC: Colorectal cancer.

When participants were stratified by gender, many differences in response patterns were observed between the gender groups. In general, females were more knowledgeable about CRC than were males, and more females knew that CRC is the cancer type associated with the most fatalities in Saudi Arabia (*P* = 0.02).

Both gender groups generally felt that CRC is preventable and curable if detected early, and that early detection of CRC through a colonoscopy is associated with high survival rates. However, both gender groups also generally reported unwillingness to undergo CRC screening.

When participants were stratified by age, many differences in response patterns were observed among the groups. For instance, the older the participant, the more likely he/she was to have correct knowledge of the colon (*P* < 0.001 between each age group), the more likely he/she was to choose to undergo a colonoscopy immediately for 2000 SR (*P* = 0.001), and the more willing he/she was to undergo CRC screening, even in the absence of symptoms (*P* = 0.006).

In general, married respondents provided more correct answers than did single respondents. For instance, married individuals were more likely to know the position of the colon (*P* = 0.033) and rectum (*P* < 0.001), as well as the function of the colon (*P* < 0.001).

The higher participants' education levels, the more likely they were to know the location of the colon and rectum and the function of the colon. For these three questions, respondents with master's and Ph.D. degrees were most likely to select the correct answer (*P* = 0.005, *P* < 0.001, *P* = 0.002, respectively). Higher education level was also associated with knowledge that CRC can develop asymptomatically, with postgraduates most likely to know this (*P* = 0.032). Finally, highly educated participants were also more likely to answer ‘yes’ when asked whether they would like to undergo CRC screening (*P* = 0.017).

## 4 Discussion

Over one-third of patients with CRC in Saudi Arabia present with late advanced stage (stage III and IV) CRC [3]. Despite a high resource availability in Saudi Arabia, inadequate knowledge and awareness among the general population represents a major barrier to the success of CRC-screening programs. Thus, in this study we aimed to assess CRC knowledge and screening awareness among the general Saudi population.

Gender was found to have an influence on knowledge level, with females generally having better knowledge of CRC. Previous studies have similarly reported that women are more knowledgeable regarding CRC symptoms, when one should undergo screening for CRC, and CRC risk factors [10,15]. However, this finding may be because females outnumbered males in our study sample and in the samples of the two abovementioned studies [10,13]. For instance, Khayyat and Ibrahim [8] found that males show higher willingness to undergo colonoscopies and participate in colon-cancer screenings, while some studies found no difference between men and women regarding knowledge and awareness of CRC [7,8,11].

We found that education level showed a positive effect on CRC knowledge. For the questionnaire item ‘can CRC develop asymptomatically?‘, the higher a participant's educational level, the more likely he/she was to answer yes, and this knowledge can directly influence screening decisions. Similarly, Khayyat and Ibrahim [8] and Al Othmani et al. [11] found that people with higher education levels tend to have better CRC-related knowledge. Furthermore, Alsmkari et al. [10] found a relationship between higher education and knowledge of CRC symptoms and risk factors, while Galal et al. [7] found having below college education level to be a negative predictor of knowledge of CRC. However, one study reported no association in this regard [15]. Considering the association observed in our and previous studies, we recommend that CRC-education efforts target individuals with lower education levels, that well-educated people be encouraged to engage in building community awareness regarding CRC, and that mandatory CRC awareness programs be implemented in schools.

Our data show that being married positively influences knowledge of CRC. A similar result was reported by Zubaidi et al. [15] who, in a survey of public awareness of CRC in Saudi Arabia, found that married respondents provide more correct answers than single respondents. Both the present study and Zubaidi et al. [15] found that married respondents are more likely to know the function and position of the colon. Furthermore, Galal et al. [7], using logistic regression, found that being unmarried is associated with lower screening uptake.

Of our population, 56% selected CRC as the cancer type associated with the most fatalities in Saudi Arabia, and 51.7% and 56.7% knew the position of the colon and rectum, respectively. However, only 21.2% were aware of the function of the colon. This indicates that the public's overall knowledge is not perfect, but is acceptable. This finding does not differ greatly from those reported in previous literature; Galal et al. [7] found poor knowledge in approximately 66.4% of their participants. To enhance community knowledge and awareness, we recommend the implementation of education programs at regular religious gatherings such as Khutba, Jumma, and Eid.

Regarding risk factors for CRC, stress was the most-chosen answer among our respondents (61.9%). In contrast, in Galal et al. [7] and Alsamkari et al. [10], similar studies to the present research, smoking was the most-selected risk factor (61.5% and 55.1%, respectively); 40.4% of our respondents chose smoking. Meanwhile, 50.3% of our respondents chose family history as a risk factor. Family history was also reported as a risk factor in Galal et al. [7] and Alsamkari et al. [10] (19.3% and 44.8%, respectively); however, in two other Saudi-Arabia-based studies most participants did not know that family history was a risk factor [15,17]. In Ahmed and Alrashidi [17], a large proportion of respondents (40.6%) believed that irritable bowel syndrome is a risk factor.

Most of our respondents (91.8%) believed that CRC is preventable, and most selected colonoscopy (72.8%) as their preferred screening method. Almost all respondents (92%) agreed that ‘early detection of CRC through colonoscopy is associated with high survival rates’; however, 65.6% answered ‘no’ when asked if they ‘would like to undergo CRC screening’. Many barriers contribute to this contradiction; if these are addressed, the rate of screening will increase. The most commonly reported barriers among our respondents were ‘fear of diagnosis/denial’ and ‘delay for administerial reasons’. Efforts should be made to remove all obstacles to CRC screening.

Several previous studies have obtained similar findings to the present results. For instance, in Alsamkari et al. [10] 86.25% of the respondents reported learning of CRC screening through social media or the internet, and colonoscopy was the preferred screening method (26%); however, 46.8% answered ‘no’ to undergoing a screening test even if they had symptoms or any risk factors. Pantel et al. [16] studied the impact of the national CRC awareness month on screening uptake, and found that although Google searches for CRC increased, no subsequent increase in screening uptake occurred. Lastly, Ahmed and Alrashidi [17] reported that 83.59% of their respondents preferred colonoscopy as a screening method and 63% answered ‘yes’ to whether it is possible to cure cancer, but that 58.59% would only undergo screening if they had symptoms.

In contrast, Khayyat and Ibrahim [8] reported that the general population's knowledge regarding screening behavior is poor, with a lack of belief in the benefit of testing (62.6%) being the primary reason their respondents were unwilling to undergo screening. Moreover, a study conducted in the Tabuk region reported that 39% of the population had insufficient knowledge regarding the benefit of early CRC screening; further, colonoscopy was the most known screening tool (31%) among this population, followed by sigmoidoscopy/fecal occult blood test (29%), and CT scan (27%) [11], and only 22% were familiar with self-screening [11]. We believe that the launching of a national screening program by an authority figure may improve the population's mindset regarding screening, which would enhance screening uptake.

Efforts to improve screening uptake must be monitored using key performance indicators relating to each step of the process. This includes acceptability, screening uptake among the population, quality and cost of screening, quality of supportive services such as pathology, and waiting times to undergo screening. Participants' data should be included in an electronic health record that affords analysis for epidemiological and quality assurance purposes. Subjects’ identities should also be linked to the national identification system, and contact with the screening authority should be conducted through a central call center to ensure confidential communication.

### 4.1 Limitations

Our study has some limitations, primarily the nature of the study design (survey-based) and the high proportion of females among our participants. Additionally, we conducted the study in a single region, which limits the generalizability of our results. Analysis of a larger sample comprising participants from all Saudi regions would afford generalization of the conclusions and provide accurate results.

## 5 Conclusions

In conclusion, there is a lack of knowledge regarding CRC among certain demographic groups in Saudi Arabia, and education and screening programs should target populations with the most limited knowledge in this regard (e.g., single males and individuals with low education levels). Moreover, elaborate efforts are needed to remove barriers to uptake of early screening for CRC, especially colonoscopies.

## Provenance and peer review

Not commissioned, externally peer-reviewed.

## Ethical approval

No personal identification information or other personal identifiers were recorded. The study protocol was approved by the ethical review board of King Saud University, Riyadh, Saudi Arabia.

## Sources of funding

No external support was received for this research.

## Author contribution

**1. Alaqel, Maram A:** Design and Literature search.

**2. Alshammari, Sulaiman A:** Data analysis and Manuscript editing.

**3. Alahmari, Shoag M:** Literature search and Manuscript preparation.

**4. Alkhayal, Nawaf K:** Data analysis.

**5. Bin Traiki, Thamer A:** Design and Manuscript editing.

**6. Alhassan, Noura S:** Concepts and Statistical analysis.

**7. Al-Obeed, Omar A:** Concepts and Manuscript review.

**8. Zubaidi, Ahmad M:** Manuscript review.

**9. Alkhayal, Khayal A:** Statistical analysis and Manuscript editing.

## Consent

Volunteered were consented.

## Registration of research studies

1. Name of the registry: Research Registry

2. Unique Identifying number or registration ID: researchregistry7228.

3. Hyperlink to your specific registration https://researchregistry.knack.com/research-registry#home/registrationdetails/6160bf2a8c1b6b001ecbd670/

## Guarantor

Shoag Alahmari.

Address: 6659 Maqboolah St., Dhahrat Laban district, Riyadh, 13751, Saudi Arabia.

Phone numbers: +966556035078.

E-mail address: shoagalahmari20@gmail.com.

## Presentation at a meeting

None.

## Declaration of competing interest

None.
